# Imidazolium salt’s toxic effects in larvae and cells of *Aedes aegypti* and *Aedes albopictus* (Diptera: Culicidae)

**DOI:** 10.1038/s41598-024-66404-3

**Published:** 2024-07-04

**Authors:** Wellington Junior da Silva, Leonardo Francisco Diel, Harry Luiz Pilz-Júnior, Alessandra Bittencourt de Lemos, Tarcísio de Freitas Milagres, Igor Luiz Gonçalves Pereira, Lisiane Bernardi, Bergmann Morais Ribeiro, Marcelo Lazzaron Lamers, Henri Stephan Schrekker, Onilda Santos da Silva

**Affiliations:** 1 Department of Microbiology, Immunology and Parasitology, Institute of Basic Health Sciences, Universidade Federal do Rio Grande do Sul, Porto Alegre, Rio Grande do Sul Brazil; 2 Faculty of Dentistry, Universidade Federal do Rio Grande do Sul, Porto Alegre, Rio Grande do Sul Brazil; 3 Laboratory of Technological Processes and Catalysis, Institute of Chemistry, Universidade Federal do Rio Grande do Sul, Porto Alegre, Rio Grande do Sul Brazil; 4 Department of Morphological Sciences, Institute of Basic Health Sciences, Universidade Federal do Rio Grande do Sul, Porto Alegre, Rio Grande do Sul Brazil; 5 Department of Celular Biology, Institute of Biological Sciences, Universidade de Brasília, Brasília-DF, Brazil

**Keywords:** Mosquito control, Histopathology, Mechanism of action, Ionic liquid, Larvicide, Midgut, Cell culture, Histology, Ionic liquids, Drug discovery and development

## Abstract

*Aedes aegypti* and *Aedes albopictus* are the main vectors of arboviruses such as Dengue, Chikungunya and Zika, causing a major impact on global economic and public health. The main way to prevent these diseases is vector control, which is carried out through physical and biological methods, in addition to environmental management. Although chemical insecticides are the most effective strategy, they present some problems such as vector resistance and ecotoxicity. Recent research highlights the potential of the imidazolium salt "1-methyl-3-octadecylimidazolium chloride" (C_18_MImCl) as an innovative and environmentally friendly solution against *Ae. aegypti*. Despite its promising larvicidal activity, the mode of action of C_18_MImCl in mosquito cells and tissues remains unknown. This study aimed to investigate its impacts on *Ae. aegypti* larvae and three cell lines of *Ae. aegypti* and *Ae. albopictus*, comparing the cellular effects with those on human cells. Cell viability assays and histopathological analyses of treated larvae were conducted. Results revealed the imidazolium salt’s high selectivity (> 254) for mosquito cells over human cells. After salt ingestion, the mechanism of larval death involves toxic effects on midgut cells. This research marks the first description of an imidazolium salt's action on mosquito cells and midgut tissues, showcasing its potential for the development of a selective and sustainable strategy for vector control.

## Introduction

Among the worldwide arboviruses transmitted by insects are Dengue, Zika, and Chikungunya, which stand out for their great epidemiological importance. The key species involved in the maintenance, replication, and transmission of these arboviruses are primarily *Aedes* mosquitoes^1^. Dengue, Zika, and Chikungunya are increasingly prevalent in various regions of the world due to the wide distribution of their vectors, including *Aedes aegypti* and *Aedes albopictus* (Diptera, Culicidae)^2^. These diseases pose a substantial challenge to global public health, characterized by elevated morbidity and mortality indicators, resulting in significant economic losses^3^.

Except for the yellow fever vaccine developed by Theiler & Smith in 1937^4^, there are currently no broadly effective vaccines against other arboviruses. Those for Dengue have some particularities, demonstrating greater efficacy against more severe clinical phenotypes, enhanced effectiveness in specific immune receptors or, variability in specific efficacy concerning viral serotype^5^. Therefore, the control of these diseases is closely related to strategies such as preventing vector-human contact, treating infections, and eliminating vector mosquitoes^6^.

Vector control involves employing physical and biological methods alongside environmental management. Although chemical insecticides are considered the most effective strategy^7^, a global survey examining vector control insecticides applied in the period of 2010 to 2019 revealed an overreliance on adulticides for residual and spatial spraying in comparison to larvicidal compounds^8^. Meier et al.^9^ asserted that employing chemical or biological products against larvae results in a reduction in the number of adult insects, leading to inhibition of reproduction. Because of their widespread use, the selective pressure on the neuronal targets of insect populations has led to the emergence of resistant populations^10,11^. Consequently, the efficacy of some insecticides has been compromised, which led to their discontinuation^9^. Alternatively, the adoption of the "pesticide treadmill" strategy, involving a mixture of several pesticides or a high-dosage regimen with predefined rotation, has been implemented^12,13^.

Significantly, even after years of insecticide application, their presence endures in the ecosystem^14^. Meier et al.^9^ provided an analysis of the ecological cost of chemical products and highlighted the excessive use and the need for higher doses to control insects. The excessive application not only damages the ecosystem but also results in environmental accumulation. Such profound use of these compounds contributes to severe environmental harm and has implications for human health^15,16^. Moreover, sustained application over time has revealed adverse effects such as water contamination, toxicity to non-target organisms, and environmental pollution^17,18^.

Hence, there is a crucial emphasis on the pursuit of novel pest control methods that minimally impact the environment while effectively managing mosquito vectors^3^. The potential toxicity of synthetic chemicals to non-target organisms^19^ makes it very difficult to develop new and more selective compounds for target insects^20^. Consequently, there is a growing interest in the study and development of "green" molecules for the control of mosquito vectors^21^.

Various imidazolium salts (IS) with biological activities, such as antimicrobial, antifungal, antitumor, and anti-inflammatory, have been identified^22^. Some studies have demonstrated the effective larvicidal action of 1-methyl-3-octadecylimidazolium chloride (C_18_MImCl) in controlling *Ae. aegypti*^23,24^ and *Culex quinquefasciatus*^25^. Pilz-Júnior et al.^25^ also confirmed the safety of using this IS through acute oral toxicity in *Wistar* rats by the up and down test (OECD 425), as well as phytotoxicity assays on *Lactuca sativa.*

In the present study, we demonstrate that IS C_18_MImCl has high selectivity for cells of *Ae. aegypti* and *Ae. albopictus* compared to human fibroblasts. Larvae of *Ae. aegypti* treated with the same salt presented cellular changes in the midgut, with modification from columnar to squamous tissue, the presence of a large number of vacuoles, and loss of microvilli from epithelial cells.

## Results

### Cytotoxicity assays

**Figure 1:**
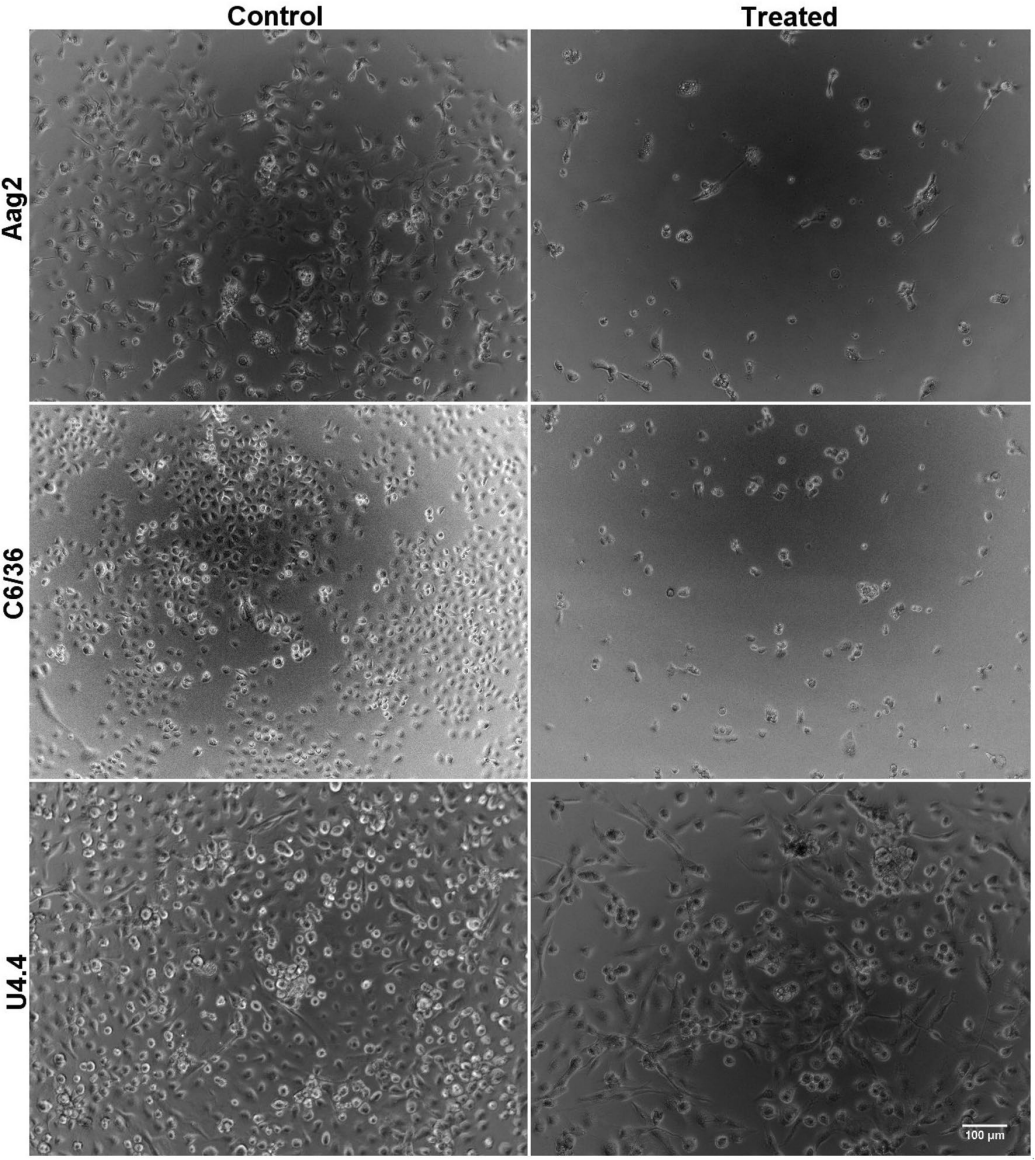
Photomicrographs showing the effect of the imidazolium salt C_18_MImCl on the three mosquito cell lines, treated for 48 h with a concentration of 0.027 µM. It is possible to observe a decrease in the number of cells in the treated group compared to the control group (culture medium only). Indicating less cell proliferation after treatment. Magnification 10×.

**Figure 2:**
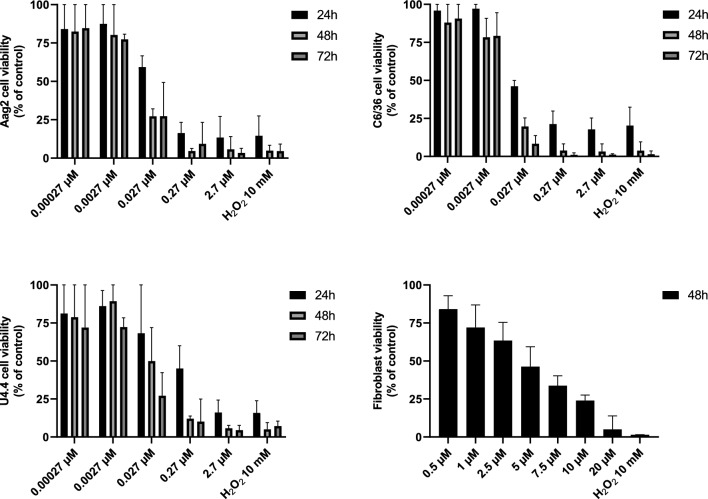
Viability assay of cells treated with C_18_MImCl. C6/36, U4.4, and Aag2 cell lines were treated with different concentrations of C_18_MImCl, and H_2_O_2_ 10 mM, and cell viability analyzed at 24, 48 and 72 h using the SRB assay. Primary human fibroblasts were treated and analyzed at 48 h. Percentage to the negative control (100%). Mean with range.

Cytotoxicity assays demonstrated that C_18_MImCl promoted a toxic effect on mosquito cell lines. In relation to the negative control group (culture medium only), treatment for 48 h with a concentration of 0.027 µM caused a decrease of 72.8, 80.3 and 50.2% in the number of cells for Aag2, C6/36 and U4.4, respectively (Fig. 1). In general, there was a dose-dependent effect on the three cell lines of *Aedes* spp. treated with the IS C_18_MImCl (Fig. 2). For the C6/36 and U4.4 strains, there were significant differences (*p* < 0.0001) between doses and time of exposure (24, 48 and 72 h), and the longer the time, lower doses were needed to promote a toxic effect. The Aag2 strain did not show differences between exposure times (*p* = 0.0125). As with the three cell lines of *Aedes* spp., fibroblasts also showed a dose-dependent response (*p* < 0.0001). For the cell lines of *Aedes* spp. and fibroblasts, the toxic concentrations ranged from 0.00027 to 2.7 µM and 0.5 to 20 µM, respectively (Table 1). The raw data is available as supplementary material.

**Table 1:** Statistical analysis for the cell lines of *Ae. aegypti* and *Ae. albopictus* treated with C_18_MImCl for 24, 48, and 72 h, as well as human fibroblasts treated for 48 h. Percentage of the negative control (100%). DF = Degrees of freedom.

2-way ANOVA		DF	F (DFn, DFd)	*P*-value
C6/36	Interaction	12	0.7521	0.6936
	Treatment	6	115.1	< 0.0001
	Time	2	22.91	< 0.0001
U4.4	Interaction	18	0.8105	0.6807
	Treatment	9	37.41	< 0.0001
	Time	2	11.59	< 0.0001
Aag2	Interaction	12	0.5159	0.8925
	Treatment	6	57.42	< 0.0001
	Time	2	4.868	0.0125
One-way ANOVA	DF	F (DFn, DFd)	*P*-value	
Fibroblasts	Treatment	7	28.05	< 0.0001

The IC_50_ values calculated for the C6/36, U4.4, and Aag2 strains from the treatments with the concentrations of 0.00027, 0.0027, 0.027, 0.27 and 2.7 µM during 24, 48 and 72 h are described in Table 2. Those IC_50_ values for fibroblasts treated with 0.5, 1.0, 2.5, 5.0, 7.5, 10 and 20 µM for 48 h are given in Table 3, together with the selectivity indexes calculated from these IC_50_ values for each cell type (Tables 2, 3).

**Table 2:** IC_50_ values calculated for *Ae. aegypti* and *Ae. albopictus* cell lines During 24, 48, and 72 h of treatment with C_18_MImCl.

		C6/36	U4.4	Aag2
24 h	IC50 *	0.01511	0.2203	0.04167
	ECF **	0.006608—0.03415	0.03031—1.416	0.01501—0.1267
	DF	15	15	15
	R^2^	0.9031	0.7701	0.8631
48 h	IC50 *	0.009012	0.03717	0.01298
	ECF **	0.004753—0.01707	0.01592—0.09635	0.004940—0.03350
	DF	15	15	15
	R^2^	0.9405	0.9	0.8752
72 h	IC50 *	0.00667	0.01528	0.01222
	ECF **	0.003605—0.01251	0.005674—0.04141	0.004842—0.03025
	DF	15	15	15
	R^2^	0.9364	0.8716	0.8944

*Doses presented in µM. **Within 95% confidence intervals, ECF: Confidence interval where the required effective concentration (EC) can be found.

**Table 3:** IC_50_ values calculated for fibroblasts after 48 h of treatment with C_18_MImCl, and selectivity indexes (SI) between mosquito cells and human fibroblasts.

IC_50_*	ECF**	DF	R^2^
9.461	3.813—27.99	18	0.8939
SI	C6/36	U4.4	Aag2
	1049.8	254.5	619.1

*Doses presented in µM. **Within 95% confidence intervals, ECF: Confidence interval where the required effective concentration (EC) can be found.

## Histopathology

**Figure 3:**
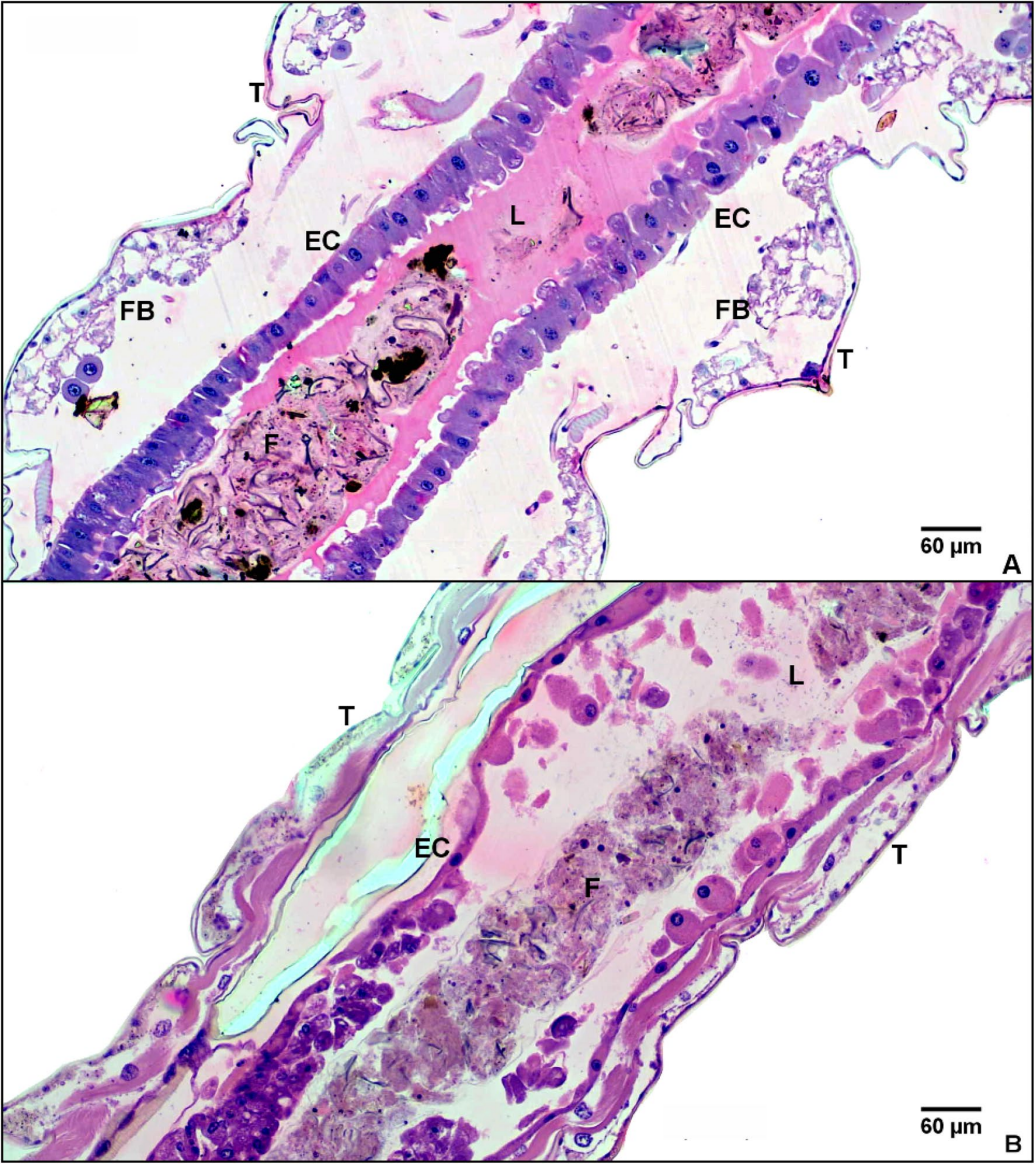
Histological analysis of internal tissues of *Ae. aegypti* larvae treated and untreated with C_18_MImCl. (**A**) Light micrograph of a negative control larva; (**B**) light micrograph of a larva treated for 12 h with LC_99_ of C_18_MImCl. The sections depicted a normal appearance in the midgut, foregut, and hindgut, peritrophic membrane, muscles, brush border, fat body, and epithelial cells. EC = Intestinal epithelial cells; L = Lumen; F = Food; T = Tegument; FB = Fat body. Hematoxylin–eosin. Magnification 10×.

**Figure 4:**
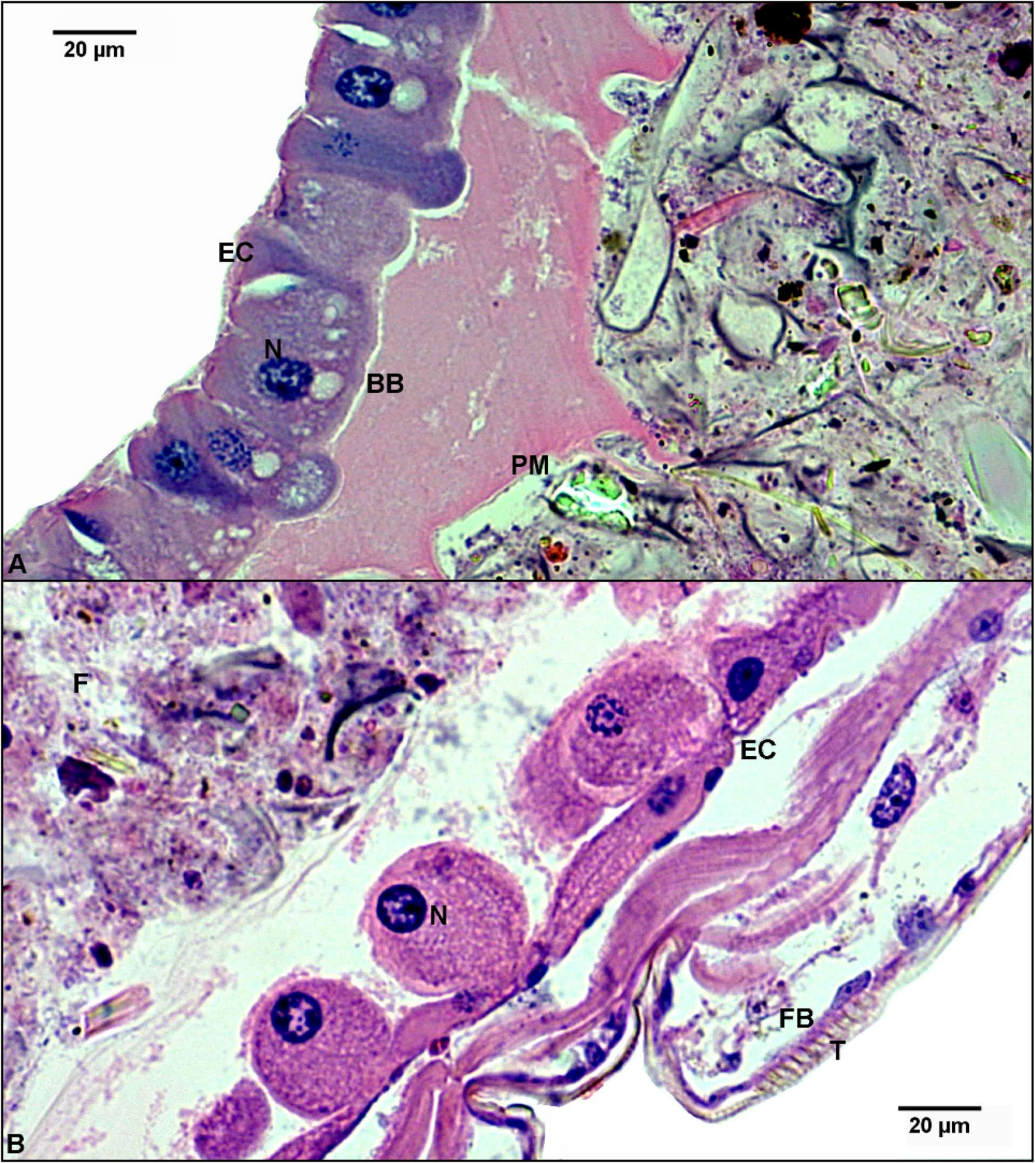
Histological analysis of internal tissues of *Ae. aegypti* larvae treated and untreated with C_18_MImCl. (**A**) Light micrograph of a negative control larva; (**B**) light micrograph of a larva treated for 12 h with LC_99_ of C_18_MImCl. The analyses revealed significant changes in the midgut epithelium, characterized by a loss of cell polarity, switching from columnar to a round shape, intense vacuolization, cell disorganization, loss of cell–cell adhesion, and loss of microvilli. Damage was observed in the fat body tissue, accompanied with larval atrophy and variations in the dimensions of the treated larvae, with the integument being closer to the intestinal epithelium. EC = intestinal epithelial cells; L = Lumen; F = Food; T = Tegument; FB = Fat body; PM = Peritrophic membrane; BB = Brush edge; N = Core. Hematoxylin–eosin. Magnification 40×.

The behavior of *Ae. aegypti* larvae, whether treated or untreated with IS, showed differences in terms of toxicity. Untreated larvae displayed typical mobility in the aquatic environment, characterized by sinuous swimming movements at various depths within the container. Histological sections depicted a normal appearance in the midgut, foregut, and hindgut, peritrophic membrane, muscles, brush border, fat body, and epithelial cells (Figs. 3A and 4A). Contrastingly, larvae treated with IS showed signs of toxicity, including reduced mobility, altered movements, and decreased response to stimuli, aligned with the criteria for evaluating larvicidal products established by the World Health Organization^26^. Histopathology analyses revealed significant changes in the midgut epithelium, characterized by a loss of cell polarity, switching from columnar to a round shape, intense vacuolization, cell disorganization, loss of cell–cell adhesion, and loss of microvilli. Damage was observed in the fat body tissue, accompanied with larval atrophy and variations in the dimensions of the treated larvae, with the integument being closer to the intestinal epithelium (Figs. 3B and 4B).

## Discussion

The findings of this study illustrate a significant selectivity of C_18_MImCl for *Aedes* spp., as evidenced by the inhibitory concentrations for each cell type. In comparison to human fibroblasts, the dosage necessary to achieve an equivalent toxic effect in the C6/36 lineage must be 1049 times higher. Similarly, concerning the U4.4 and Aag2 cells, the doses necessary to induce a comparable toxic effect in human fibroblasts must be 254 and 619 times higher, respectively.

Until now, the research that explored the impact of IS on different cell types is limited. Martins et al.^27^ investigated the toxic effect of different IS molecules against *Leishmania amazonensis* and certain human cells. In peripheral blood mononuclear cells (PBMC), almost all IS caused dose-dependent cytotoxicity. Assessing the CC_50 PBMC_/_IC50 *Leishmania*_ ratio, C_18_MImCl showed low cytotoxicity for PBMC with a selectivity index of 15.35. The authors also performed a hemolytic assay, demonstrating that human erythrocytes were less susceptible to the cytotoxic effect of IS, with significant hemolysis observed only at concentrations ≥ 30 μM, yielding a selectivity index ≥ 30^27^.

The work conducted by Pilz-Júnior et al.^25^ also highlighted the low cytotoxicity of C_18_MImCl, revealing the effectiveness of IS in controlling *Culex quinquefasciatus* larvae with minimal impact on non-target-organisms. In the V79 cell line (murine fibroblasts), C_18_MImCl demonstrated biocompatibility even at doses up to 5 µM, with a CC_50_ of 12.8 µM. For HaCat cells (keratinocytes), the estimated CC_50_ for C_18_MImCl was 18.1 µM^25^. Additionally, Pilz-Junior et al.^24^ demonstrated bacterial modification in the microbiota of larvae treated with the same IS.

In this study involving *Ae. aegypti* treated with C_18_MImCl, various toxic effects were observed. These included alterations in the midgut epithelium, featuring a transition from columnar to squamous tissue, the presence of vacuoles, and loss of microvilli in the epithelial cell layer. As far as we are aware, this is the first study demonstrating the histopathological effect an IS on mosquito larvae. Interestingly, these toxic effects are comparable with those found in larval tissues treated with bacteria and fungi^20,28,29^. These effects differed significantly from those observed when mosquito’s species were treated with chemical insecticides like the organophosphate temephos. Several studies utilizing natural bioactive compounds have demonstrated similar alterations in midgut cells like the IS studied^30^.

Histological analysis of *Ae. aegypti* and *Anopheles stephensi* treated with polysaccharides from *Bacillus licheniformis* revealed muscle damage, abdominal atrophy, and lesions in the midgut^28^. Treatment with a limonoid compound from *Penicillium oxalicum* induced damage to microvilli, peritrophic membranes, and epithelial cells of *C. quinquefasciatus* larvae^29^. In *An. stephensi* larvae treated with fraction 1 (F1-emodin) of the mycelial extract of *Aspergillus terreus*, the midgut emerged as the most affected tissue, displaying disruption in the peritrophic membrane and the intestinal epithelium. Additionally, cellular vacuolization, muscle damage, and disorganization of the brush border were observed. For *Ae. Aegypti* larvae, histological changes encompassed the gastric cecum, hindgut, and nerve ganglia, alongside midgut collapse accompanied by vacuolation of epithelial cells^20^.

Consistent with the findings outlined in this study work, Vivekanandhan et al.^31^ conducted a histopathological analysis of *C. quinquefasciatus* larvae exposed to *Beauveria bassiana*-28 extract. Their results similarly demonstrated damage to the cells of the intestinal epithelium, accompanied by vacuolation, destruction of the cell membrane, infiltration of the luminal contents into the muscle tissue and cell damage. Additionally, disorganization of the fat body was observed.

The lesions observed in the histopathological analysis of this study suggest that the IS-induced larval death process is linked to the disruption of intestinal homeostasis, potentially leading to various death pathways: (a) interference in nutrient absorption due to the loss of microvilli; (b) damage to the intestinal cell membranes, enabling infiltration of luminal contents into the hemocoel, which may result in (b-1) septicemia, (b-2) damage to muscle cells causing paralysis of movement, or (b-3) entry of IS molecules into the fat body, destabilizing trophocytes and oenocytes and interference in larval metabolism.

It is noteworthy that there is no class of chemical larvicide with the properties of the presented imidazolium salt, which does not present phytotoxicity or toxicity to mammals^25^. As described in the work of Goellner et al.^23^, C_18_MImCl showed a residual effect with 95% and 80% larval mortality for 36 and 78 days, respectively, after being dried under environmental conditions for at least two months and then dissolved in water for use.

In conclusion, the IS C_18_MImCl exhibits notable selectivity for *Ae. aegypti* and *Ae. albopictus* cells in comparison to human fibroblasts. The mechanism of death appears to be associated with toxic effects of C_18_MImCl in larvae’s midgut after its ingestion from water. These findings underscore the potential of this IS as a promising agent for addressing the current challenges in *Aedes* control. Additionally, the importance of conducting further investigations with diverse approaches is emphasized to develop new strategies for effective vector control.

## Material and methods

### Imidazolium salt

1-Methyl-3-octadecylimidazolium chloride (C_18_MImCl; purity > 99%; 371.04 g/mol) was purchased at CJC China Jie Chemical and recrystallized.

### Larvae, reagents, and cell culture

The larvae of *Ae. aegypti* (Rockefeller strain) come from a colony maintained under controlled conditions of temperature (± 28 ºC) and humidity (± 80%) in the Laboratory of Arthropod Vectors of the Department of Microbiology, Immunology, and Parasitology at Institute of Basic Health Sciences—ICBS/UFRGS.

Cell line of *Aedes aegypti* Aag2 –derived from embryonic tissue^32,33^ and *Ae. albopictus* U4.4 –derived from newly hatched axenic larvae^34^, were received from the University of Brasilia. *Aedes albopictus* C6/36 ATCC® CRL-1660™ -derived from newly hatched axenic larvae^34,35^ was obtained from the Cell Bank of Rio de Janeiro, Brazil. Two different *Ae. albopictus* cell lines were used to evaluate the effectiveness of C_18_MImCl and verify whether the same effect occurred in both strains, and thus provide robustness for the analysis and interpretation of the results presented. Primary fibroblasts were donated by the Laboratory of Cell Biology of the Institute of Basic Health Sciences at UFRGS. All cell lines were cultured in Dulbecco's modified Eagle's medium (DMEM – Gibco, Thermo Fischer Scientific, Massachusetts, USA) with low levels of glucose, supplemented with 1% non-essential amino acids (NEAA – Gibco, Thermo Fischer Scientific, Massachusetts, USA), maintained at 28 °C in a 5% CO_2_ incubator. Primary human fibroblasts were cultured in DMEM medium at 37 °C and maintained in a 5% CO_2_ incubator.

### C_18_MImCl activity in human and mosquito cells

Cell viability was determined by measuring protein content with the sulforhodamine B assay (SRB)^36^. Mosquito cell lines (6 × 10^3^ cells/well) and human fibroblasts (2 × 10^3^ cells/well) were incubated overnight in DMEM culture medium. The IS C_18_MImCl was diluted in ultrapure water (Milli-Q) and treatments were performed at concentrations ranging from 0.00027 to 2.7 µM for 24, 48, and 72 h for cell lines of *Aedes* spp., and concentrations between 0.5 to 20 µM for 48 h for fibroblasts. Hydrogen peroxide (10 mM) was used as a positive control. After the incubation period, the cell monolayers were fixed with 10% trichloroacetic acid, washed with running water, and kept overnight at room temperature for drying. Afterward, staining was performed for 30 min with 0.4% SRB dye diluted in 1% acetic acid, excess dye was removed by repeated washing with 1% acetic acid. Protein-bound dye was dissolved in 10 mM Tris base solution for optical density (OD) determination. Next, absorbance was read at a wavelength of 560 nm in a Multiskan GO microplate reader (Thermo Fisher Scientific).

The concentration of IS that caused the death of 50% of cells was determined by nonlinear regression analysis (curve fit; hill slope = 1; F = 50) at confidence level of 95%, using the equation: Treatment = Log (treatment) vs. response—Find ECanything Least squares fit. For cell lines of *Aedes* spp., IC_50_ values were calculated at 24, 48, and 72 h of exposure, the one for fibroblasts at 48 h. The selectivity index (SI) of C_18_MImCl was determined by the equation: IC_50_ against fibroblasts/IC_50_ for *Aedes* spp. cells.

### Histopathological analysis of larvae exposed to C_18_MImCl

The histopathological analysis was performed according to Lemos et al.^37^. Two groups of larvae were used in this bioassay: 25 larvae of the 3rd and 4th instars of *Ae. aegypti* maintained only in 150 mL of distilled water (control group), and 25 larvae treated with 150 ml of C_18_MImCl at CL_99_—18.862 µg/ml^23^. The 12-h treatment time was determined from signs of toxicity observed during the exposure of the groups, mainly, slowness and change in movements, and decreased response to stimuli^26^. After exposure, the samples were fixed in Karnovsky's solution^38^ for 24 h at 4 °C, and washed with 70% alcohol. Dehydration was performed by immersing larvae in a series of increasing concentrations of ethanol: 70% 10 min, 95% 15 min, 100% 20 min, and 100% 30 min. Then the samples were soaked in a resin solution (Leica Historesin Embedding Kit) at room temperature. Embedding was performed in 6 × 8 mm polyethylene molds. The molds were kept at 40 °C for at least 12 h to ensure complete polymerization of the resin. Longitudinal sections of 3 μm were made using a Lupetec MRP 2015 semi-automatic rotary microtome with disposable blades. For each resin block, 10 to 15 microscopy slides were prepared, on which six sequential sections were fixed. These slides were stored in an oven at 30 ± 2 °C for 24 h. For the analysis, slides were stained with Harris hematoxylin and eosin. The sections were examined and photographed using a Nikon Eclipse Si microscope coupled with a Digital Sight 1000 camera in conjunction with the Capture 2.3 program.

## Statistical analysis

Viability assays were performed in quadruplicate and repeated three times. Data were submitted to the Kolmogorow-Smirnow test to assess normality. Variables with normal distribution were compared by analysis of variance, repeated measures or one-way ANOVA, and Tukey's multiple comparisons test. Significant differences were considered when *p* value < 0.0001. The GraphPad Prism 8.3.0 (GraphPad Software, Boston, Massachusetts, USA) program was used to carry out the analyses.

### Supplementary Information

Supplementary Information.

## Data Availability

All data generated or analyzed during this study are included in this published article [and its supplementary information files].
